# A Novel AgNPs/Sericin/Agar Film with Enhanced Mechanical Property and Antibacterial Capability

**DOI:** 10.3390/molecules23071821

**Published:** 2018-07-23

**Authors:** Yejing Wang, Rui Cai, Gang Tao, Peng Wang, Hua Zuo, Ping Zhao, Ahmad Umar, Huawei He

**Affiliations:** 1 College of Biotechnology, Southwest University, Beibei, Chongqing 400715, China; yjwang@swu.edu.cn (Y.W.); cairui0330@email.swu.edu.cn (R.C.); modelsums@email.swu.edu.cn (P.W.); 2 State Key Laboratory of Silkworm Genome Biology, Southwest University, Beibei, Chongqing 400715, China; taogang@email.swu.edu.cn (G.T.); zhaop@swu.edu.cn (P.Z.); 3 College of Pharmaceutical Sciences, Southwest University, Beibei, Chongqing 400715, China; zuohua@swu.edu.cn; 4 Chongqing Engineering and Technology Research Center for Novel Silk Materials, Southwest University, Beibei, Chongqing 400715, China; 5 Department of Chemistry, College of Science and Arts and Promising Centre for Sensors and Electronics Devices, Najran University, P.O. Box 1988, Najran 11001, Saudi Arabia; umahmad@nu.edu.sa

**Keywords:** silk sericin, agar, silver nanoparticles, antimicrobial activity

## Abstract

Silk sericin is a protein from a silkworm’s cocoon. It has good biocompatibility, hydrophilicity, bioactivity, and biodegradability. However, sericin could not be used in biomedical materials directly because of its frangible characteristic. To develop multifunctional sericin-based materials for biomedical purposes, we prepared a sericin/agar (SS/agar) composite film through the blending of sericin and agar and repetitive freeze-thawing. Then, we synthesized silver nanoparticles (AgNPs) in situ on the surface of the composite film to endow it with antibacterial activity. Water contact angle, swelling and losing ratio, and mechanical properties analysis indicated that the composite film had excellent mechanical property, hydrophilicity, hygroscopicity, and stability. Scanning electron microscopy and X-ray photoelectron spectroscopy analysis confirmed the successful modification of AgNPs on the composite film. X-ray powder diffraction showed the face-centered cubic structures of the AgNPs. This AgNPs modified composite film exhibited an excellent antibacterial capability against *Escherichia coli* and *Staphylococcus aureus*. Our study develops a novel AgNPs/sericin/agar composite film with enhanced mechanical performance and an antimicrobial property for potential biomedical applications.

## 1. Introduction

Recently, it appears promising to combine the outstanding properties of natural protein and polysaccharides for biomedical applications [1]. As a natural macromolecular protein, silk sericin (SS) is composed of 25% of the cocoon that is produced by the silkworm [2,3]. Sericin is composed of 18 different amino acids [4]. As sericin has a content of serine that is about 33.43% [5], it is considered as a natural moisturizing factor for the human skin due to its excellent moisture retention capacity [6]. Sericin is not expensive, is readily available, is biocompatible, and biodegradable [7,8,9]. Sericin is a biologically active substance in wound dressings due to its diverse activities such as ROS clearance, anti-tyrosinase, and its immunomodulatory capacity [10,11]. In addition, sericin can promote skin keratinocytes and fibroblasts adhesion and proliferation [12,13,14,15], especially for cell growth and migration [16,17], which makes it favorable for wound dressing and tissue engineering applications [18,19,20,21]. Sericin can efficiently promote wound healing by accelerating collagen deposition and the re-epithelialization of skin tissue [22]. However, sericin has a large amount of disordered structures [23,24], resulting in its poor mechanical performance [25]. Thus, crosslinking, blending, or copolymerizing with other substances is often applied to overcome the brittleness of sericin [26].

Agar is a kind of unbranched polysaccharide that is produced by seaweed [27]. It can spontaneously form gels at a very low concentration. Hence, it is widely applied in the food industry, the pharmaceutical industry, and in cosmetics [28,29]. In addition, agar is applied in biomaterials for its high mechanical strength and biocompatibility [30]. It is promising to combine the outstanding properties of sericin and agar to produce a novel composite material with suitable mechanical properties and good biocompatibility for biomedical applications such as tissue engineering and wound healing.

Surface immobilization with an antimicrobial agent is a common method to prepare antibacterial materials. Silver nanoparticle (AgNP) has broad-spectrum antimicrobial activities [31,32,33,34] and rarely leads to drug resistance [35]. Also, AgNP has good cytocompatibility [36,37]. AgNP has an anti-inflammatory effect which is beneficial for wound healing [38,39]. AgNPs are commonly modified on materials after synthesis. The traditional procedure is too complicated and time-consuming to be abandoned [40,41,42]. The biosynthesis of AgNPs is a safe and environmentally friendly method and has received increasing attention [43,44]. However, biosynthesis is too difficult to ensure the in situ synthesis of AgNPs. UV irradiation is usually used to in situ synthesize AgNPs on the surface of materials [45,46,47]. Here, we blended sericin and agar to prepare a SS/agar film through freeze-thawing for four cycles. Then, the AgNPs were immobilized on the composite film for their antimicrobial purpose.

## 2. Results and Discussion

**Figure 1:**
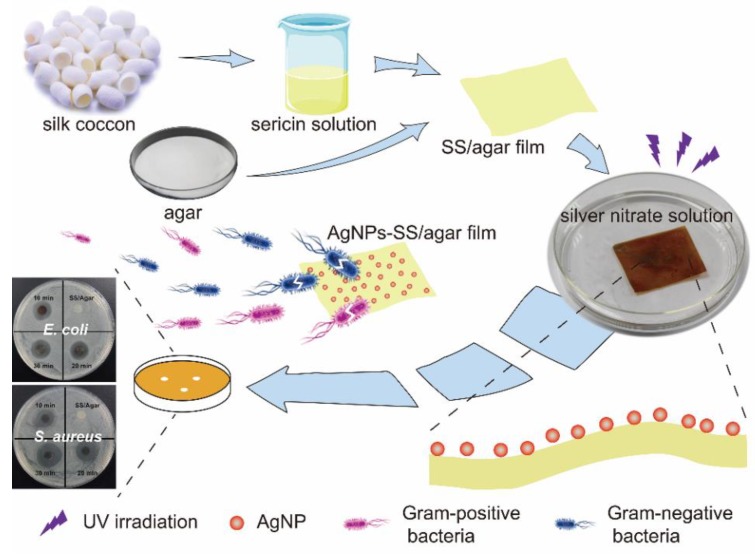
A diagram of the fabrication and antimicrobial test of the AgNPs/SS/agar film.

Here, we prepared a SS/agar film through blending without an extra cross-linking agent. Sericin was extracted into water through autoclave. A high temperature (60 °C) promoted the blending of sericin and agar. As the temperature decreased, the mixture gelled to form a novel SS/agar film. UV-assisted synthesis is a facile and green approach to synthesize AgNPs in situ on the surface of a material without chemical cross-linking reagents. Here, UV irradiation was used to promote the synthesis of AgNPs. Then, the antimicrobial property of the composite film was evaluated by inhibition zone and bacterial growth assays. The diagram of the fabrication and antimicrobial analysis of AgNPs/SS/ agar film are shown in Figure 1.

**Figure 2:**
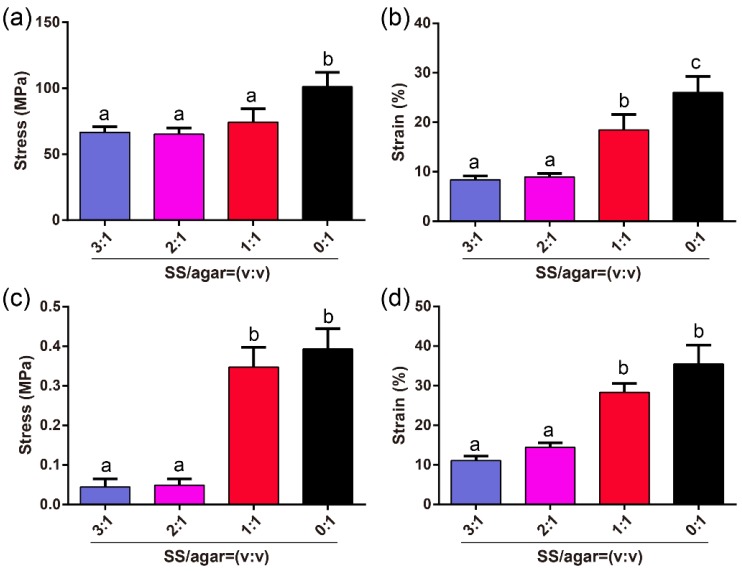
Mechanical properties of the SS/agar films with different ratios (SS: agar = 3:1, 2:1, 1:1, 0:1). The stress of the SS/agar film in a dry state (**a**) and wet state (**c**); The strain of the SS/agar film in a dry state (**b**) and wet state (**d**). For statistical analysis, the bars that are labeled with different lowercase letters (a, b, c) are significantly different, and the same letter indicates no significant difference (*p* < 0.05).

To determine the effects of the agar contents on the performance of the SS/agar film, the mechanical performance of the SS/agar films with different agar contents were determined. The stress and strain of the SS/agar film increased with increasing agar content in the dry state (Figure 2a,b), respectively. The sericin film failed to test as it was too brittle. The pure agar film had the highest stress and strain. The stress and strain of the SS/agar film (1:1) were 74.3 ± 10.6 MPa and 19.4 ± 4.6%, respectively, which were the highest of the tested films. Figure 2c,d showed a similar tendency in a wet state. The result suggested that agar could significantly improve the rigidity and flexibility of the sericin film. In a wet state, the strain of the SS/agar film (1:1) was 30.3 ± 1.7%, which was higher than that of the film in a dry state, indicating that SS/agar film had better flexibility in the wet state than in the dry state. Our result suggested that the prepared SS/agar film had good rigidity and flexibility, which may have a potential application in biomaterials such as wound dressing and tissue engineering [48,49,50].

**Figure 3:**
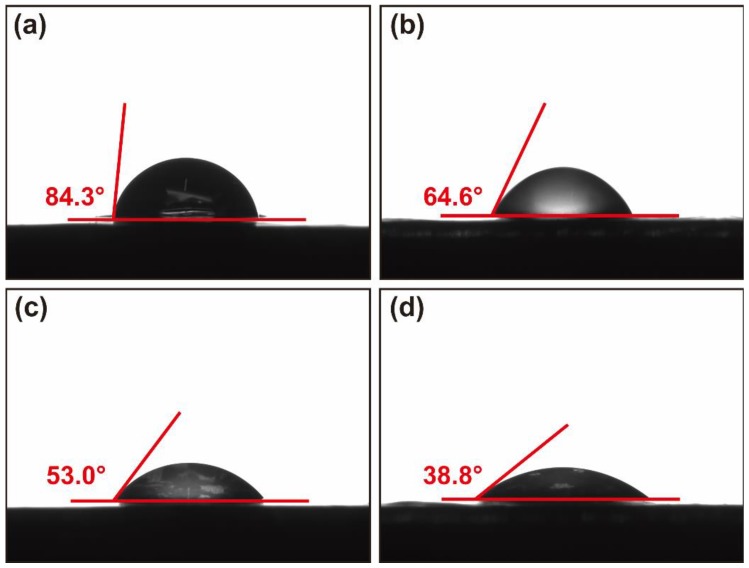
The water contact angle of the SS/agar films. SS: agar = 0:1 (**a**), SS: agar = 1:1 (**b**), SS: agar = 2:1 (**c**), and SS: agar = 3:1 (**d**).

The water contact angle is an important feature of a material’s surface hydrophobicity. A water contact angle that is less than 90° indicates that the material is hydrophilic. The agar film had a water contact angle of 84.3°. Increasing sericin’s content resulted in a decrease of the water contact angle from 84.3° to 38.8° (Figure 3), indicating that sericin could significantly improve the hydrophilicity of the agar film. This is related to the hydrophilicity of sericin as it contains a great deal of hydrophilic groups. 

**Figure 4:**
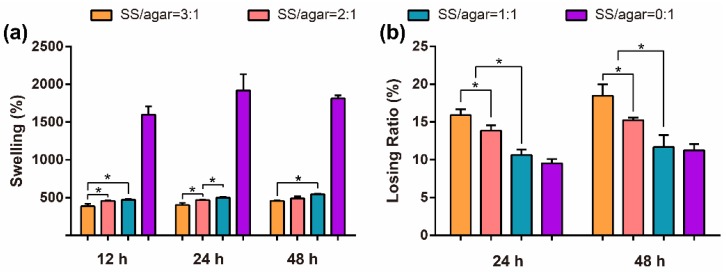
The swelling (**a**) and losing ratio (**b**) of the different SS/agar films. * indicates *p* < 0.05.

Swellability indicates the water absorption capacity. The pure agar film had a swelling ratio of 1599.1 ± 84.7% after 12 h (Figure 4a), indicating its good hygroscopicity [27]. The swelling ratio of the SS/agar film increased with the increase of agar content, suggesting that agar could improve the swellability of the SS/agar film. The SS/agar film (1:1) had a swelling ratio of 472.3 ± 9.2% after 12 h (Figure 4a), suggesting that it had good hygroscopicity. The excellent hydrophilicity and swellability are beneficial to the SS/agar film as a wound dressing to keep a moisture microenvironment around the wound interface to promote wound healing.

The losing ratio is an indicator of a material’s stability. The pure agar film had the lowest losing ratio in all of the films. The losing ratio of the SS/agar film decreased with the increase of agar content (Figure 4b), suggesting that agar could improve the stability of the SS/agar film. The SS/agar film (1:1) had a losing ratio of 10.6 ± 0.8% after 24 h (Figure 4b), indicating that it had good stability.

**Figure 5:**
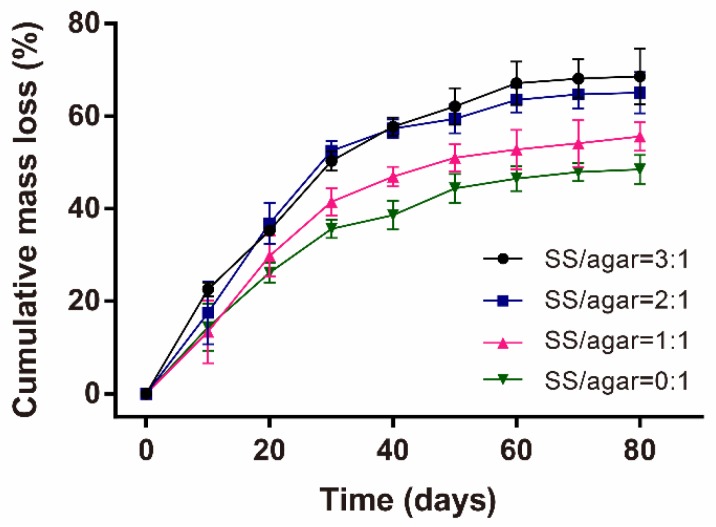
The mass loss of the SS/agar films.

Stability is very important for a wound dressing. The mass loss of the SS/agar film in the PBS buffer (pH 7.4) was used to evaluate its stability. After 80 days, the agar film and the SS/agar film (1:1) lost about 48.5% and 55.6% of the initial mass (Figure 5), respectively. The mass loss ratio of the SS/agar film increased with increasing sericin content. Our result indicated the excellent stability of the SS/agar film.

Taken together, the results suggested that the SS/agar film (1:1) had good mechanical performance, excellent hydrophilicity, hygroscopicity, and stability. As a result, the SS/agar film (1:1) was selected for the following experiments.

**Figure 6:**
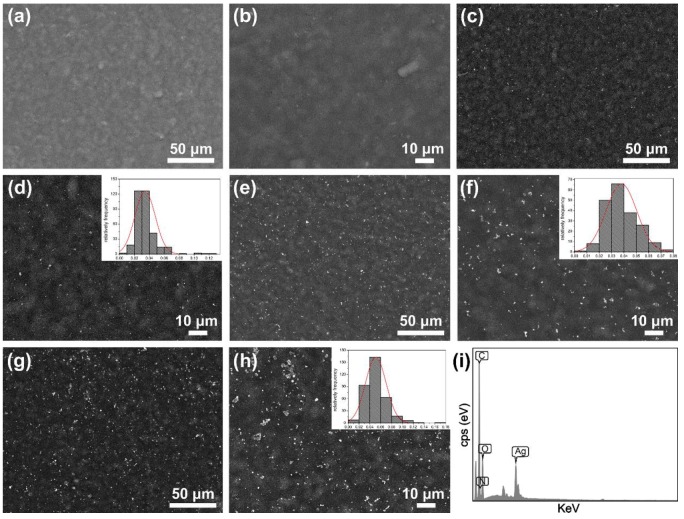
The surface morphology of the different films. The SS/agar film (**a**,**b**) and AgNPs/SS/agar films after UV irradiation for 10 min (**c**,**d**), 30 min (**e**,**f**), and 60 min (**g**,**h**), respectively; inset in (**d**,**f**,**h**): the size distribution of the AgNPs. (**i**) Elemental composition analysis of the AgNPs/SS/agar film by EDS.

The surface morphologies of the composite films were characterized by scanning electron microscopy (SEM). As shown in Figure 6a,b, the SS/agar film had a smooth and uniform surface without any defects, indicating that the agar and sericin were well blended. Some small dots appeared on the SS/agar film after UV irradiation for 10 min (Figure 6c,d), indicating the successful synthesis of AgNPs. The synthesized AgNPs had a size range of 20–50 nm (Figure 6d). Increasing the irradiation time promoted AgNPs synthesis (Figure 6e–h). After UV irradiation for 30 min and 60 min, most of the AgNPs had a size range of 20–60 nm and 20–80 nm, respectively (Figure 6f–h). This result suggested that UV irradiation time could regulate AgNPs synthesis. EDS spectrum showed a featured peak of silver element (Figure 6i), indicating the existence of silver on the AgNPs/SS/agar film. The other featured peaks, such as carbon, nitrogen, and oxygen, were suggested to be from the sericin and agar of the film (Figure 6i).

**Figure 7:**
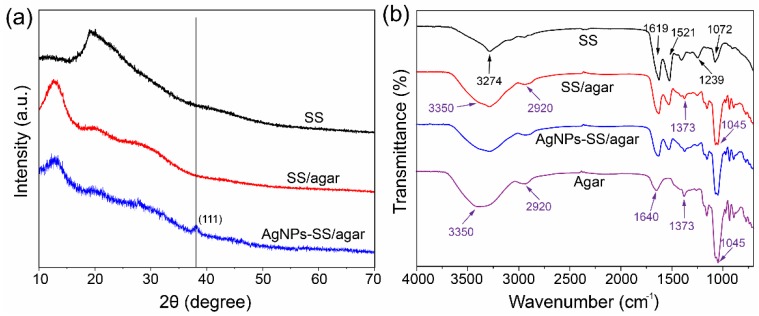
The characterization of different films. (**a**) XRD spectra; (**b**) FT-IR spectra.

X-ray powder diffraction (XRD) was performed to characterize the composite films, as shown Figure 7a. The peak that was located at 19.4° and 13.9° could be assigned to the featured pattern of sericin [51] and the crystalline diffraction of agar [28,30], respectively. The AgNPs/SS/agar film had a significant XRD peak at 38.2°, indicating that the synthesized AgNPs had the crystal planes (111) structure [52,53]. The excellent crystalline structure of the AgNPs implied its efficient antimicrobial activity.

The FT-IR spectra showed the featured peaks of sericin at 1619 cm^−1^ and 1521 cm^−1^ (Figure 7b), which are assigned to the amide I and II of the β-sheet structure [54,55]. The specific bands of agar at 3350 cm^−1^ and 2920 cm^−1^ were associated with the stretching vibration of the O-H and C-H bonds, respectively. The bands at 1640 cm^−1^ and 1373 cm^−1^ were assigned to the stretching vibration of the peptide bonds. The bands at 1072 cm^−1^ and 1045 cm^−1^ were related to the vibration of the C-O bond [28]. After blending with agar, the amide I, II, and III bands of sericin did not change, indicating that the blending did not obviously affect sericin’s structure. In addition, after the AgNPs modification on the surface of the SS/agar film, the amide bands of sericin did not change, suggesting that the modification had no effect on the SS/agar film’s structure.

**Figure 8:**
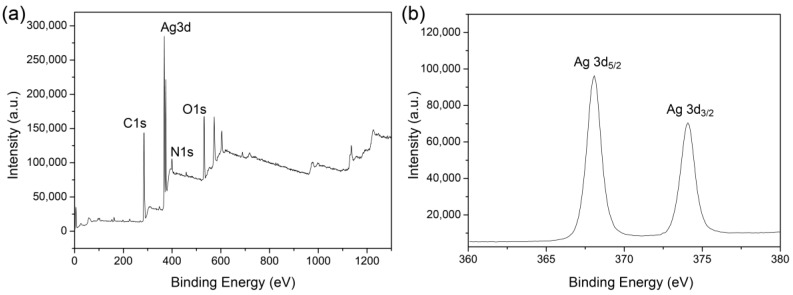
XPS spectra of the AgNPs/SS/agar film (**a**) and the deconvolution of the Ag (3d) peak (**b**).

X-ray photoelectron spectroscopy (XPS) was carried out to reveal the chemical valence of the synthesized silver. Ag (3d_5/2_) and Ag (3d_3/2_) had the binding energies of 368.08 eV and 374.08 eV (Figure 8), respectively, indicating the formation of Ag^0^ [56,57]. Further, the Ag (3d) peak was analyzed by a deconvolution algorithm. Ag, Ag_2_O, and AgO have the binding energies of 368.5 eV, 368.3 eV, and 367.7 eV, respectively. Our result suggested that the synthesized AgNPs were composed of about 95% Ag^0^, 1% Ag_2_O, and 4% AgO.

**Figure 9:**
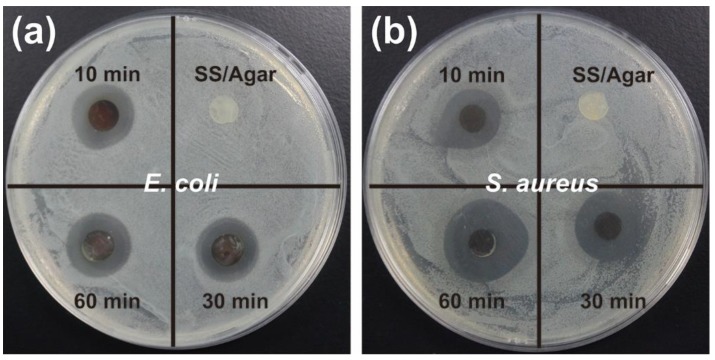
The inhibition zone assay of the SS/agar and AgNPs/SS/agar films. (**a**) *E. coli* and (**b**) *S. aureus*.

*E. coli* and *S. aureus* were chosen as the model of Gram-negative and Gram-positive bacteria to assess the bactericidal activities of the AgNPs/SS/agar film. The inhibition zone assay showed that no inhibition rings appeared in the presence of the SS/agar film, either on *E. coil* or *S. aureus* agar plates. Whereas, the AgNPs/SS/agar films formed obvious inhibition rings on *E. coil* or *S. aureus* agar plates (Figure 9), indicating the excellent antimicrobial activity of the composite film. We measured the diameter of the inhibition rings that were formed, and these are listed in Table 1. There was a statistical difference between the groups for *S. aureus*, but there was no significant difference between the groups for *E. coli*. This result showed that the diameter of the inhibition ring increased with the increase of UV irradiation time, indicating that increasing the UV irradiation time promoted the synthesis of AgNPs and enhanced the antibacterial capability of the AgNPs/SS/agar film on *E. coli* or *S. aureus*. 

**Figure 10:**
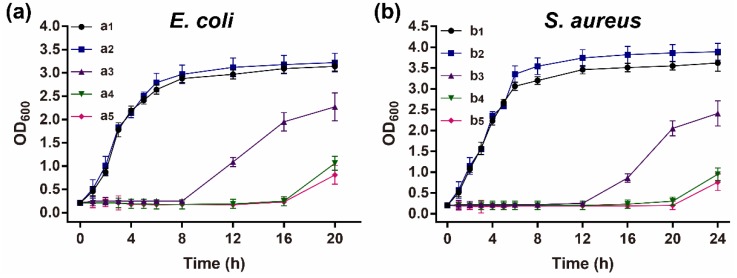
Bacterial growth assay. (**a**) *E. coli* and (**b**) *S. aureus*. a1, b1, control; a2, b2, SS/agar film; a3–b5, AgNPs/SS/agar films with UV irradiation for 10 min (a3, b3), 30 min (a4, b4), and 60 min (a5, b5).

A bacterial growth assay was performed to further measure the antibacterial activity of the composite film. The bacterial growth showed a similar tendency in the presence of the SS/agar film with that of the control. Nevertheless, in the presence of the AgNPs/SS/agar films, the bacterial growth was obviously inhibited, either for *E. coil* or *S. aureus* (Figure 10). This result was in good agreement with that of the inhibition zone assay, suggesting that the AgNPs/SS/agar film had excellent antimicrobial activity against *E. coil* and *S. aureus*. The AgNPs/SS/agar film with UV irradiation for 60 min showed the best inhibitory effect on the bacterial growth among all of the tested films, either for *E. coli* or *S. aureus*. Given the results of the inhibition zone and bacterial growth assay, the AgNPs modified SS/agar film with UV irradiation for 60 min had the best inhibitory effect on bacteria.

**Figure 11:**
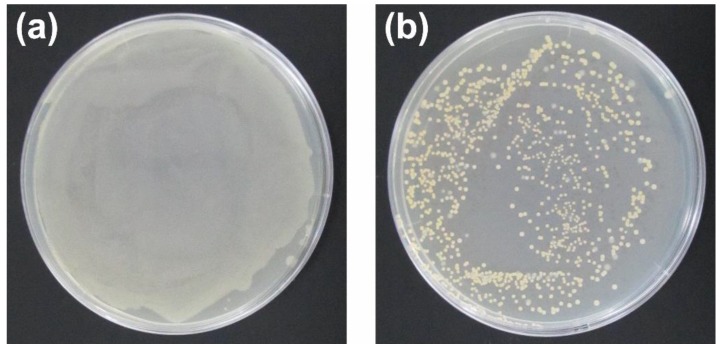
A comparison of the bacterial colony numbers that were treated by the AgNO_3_/SS/agar film (**a**) and the AgNPs/SS/ agar film (**b**).

To compare the antibacterial property of Ag (I) ions and AgNPs on *E. coli*, a bacterial colony counting assay was performed. The result showed that the number of bacterial colonies that were treated by the AgNO_3_/SS/agar film was much more than that of the bacterial colonies that were treated by the AgNPs/SS/agar film (Figure 11), indicating that the AgNPs had a more significant antibacterial effect than the Ag (I) ions on *E. coli*.

## 3. Materials and Methods 

### 3.1. Materials and Reagents

Silkworm cocoons were kindly provided by the State Key Laboratory of Silkworm Genome Biology, Southwest University, China. In our laboratory, we reared a practical strain of silkworm 872 with fresh mulberry leaves at 25 °C under a photoperiod of 12 h light and 12 h dark and 75% relative humidity. We collected the cocoons for sericin preparation. Agar and AgNO_3_ (99.99%) were products of Aladdin (Shanghai, China). Ultrapure water that was used in the experiment was prepared by a Milli-Q system (Millipore, Burlington, MA, USA).

### 3.2. Fabrication of the SS/Agar Film

Sericin was obtained from the silk cocoon as previously reported [58]. The silk cocoons were sliced and autoclaved at 121 °C and 103.4 kPa for 15 min to extract sericin into the water. The insoluble silk fibroin was removed by filtration. The sericin solution was lyophilized to sericin powder. The sericin powder was dissolved in water to become 2% (*w*/*v*) sericin solution at 60 °C. The agar was dissolved in water with continuous stirring at 85 °C. Different volume ratios of sericin (2%, *w*/*v*) and agar (2%, *w*/*v*) were well blended at 60 °C for 15 min and were then air-dried at 37 °C to become the SS/agar film.

### 3.3. AgNPs Synthesis

AgNPs synthesis with the assistance of UV irradiation is a popular method [59,60]. First, the SS/agar film was soaked in AgNO_3_ solution (50 mM) at 25 °C, then the film and AgNO_3_ were exposed to UV irradiation for 10, 30, or 60 min. Ag^+^ was reduced to AgNPs in situ on the SS/agar film with the assistance of UV light. Finally, the AgNPs modified SS/agar film was removed from the AgNO_3_ solution and was air-dried at 37 °C.

### 3.4. Mechanical Property

The mechanical performance of the SS/agar films were evaluated as the stress and strain that was measured on an AG-Xplus universal machine (Shimadzu, Kyoto, Japan). Each film was sliced into a long strip (5.5 cm × 1 cm). The strip’s thickness was determined by an Olympus microscope. Each film was measured at least 10 times and the average value was converted to the stress and strain [61].

### 3.5. Wettability

The film’s wettability was assessed by the water contact angle which was measured on a Krüss DSA100 system (Hamburg, Germany) at 25 °C. To do this, 4 μL of water was dropped on the surface of the film. The complete process of water absorption at five different positions was recorded for each film. The water contact angle was calculated and averaged from five independent measurements.

### 3.6. Swelling and Losing Ratio

The swelling ratio was determined to measure the film’s hygroscopicity, as described by Mandal et al. [62]. After weighing, the dried films (3 cm × 3 cm) were immersed in 10 mL of PBS buffer (pH 7.4) for 12–48 h at 37 °C. Then, the films were taken out from the PBS buffer and were weighed after the removal of the extra water on the surface of the films. The mass of the dried and swollen films were weighed as *W*_1_ and *W*_2_, respectively. Each film was measured at least five times. The swelling ratio was calculated using the Formula (1):

(1) $$\;S\left(\%\right)=\frac{W_{2}-W_{1}}{W_{1}}\times 100\%\;$$

The losing ratio was measured to determine the stability of the film. Each sample (3 cm × 3 cm) was immersed in 10 mL of PBS buffer (pH 7.4) for 24–48 h at 37 °C. Then, the film was separated from the PBS buffer and was dried at 65 °C. The initial mass of the SS/agar film and the residual mass of the film after the treatment were weighed as *W*_3_ and *W*_4_, respectively. Each film was measured in triplicate. The losing ratio was determined using the Formula (2):

(2) $$\;L\left(\%\right)=\frac{W_{3}-W_{4}}{W_{3}}\times 100\%\;$$

### 3.7. Mass Loss

The SS/agar films (3 cm × 3 cm) were soaked in 30 mL of PBS buffer (pH 7.4) at 37 °C after drying at 65 °C for 24 h. The initial mass of the film was weighed as *W*_5_*.* The PBS buffer was substituted daily to keep it fresh. At different intervals, the films were dried at 65 °C for 24 h after separation from the PBS buffer and were then weighed as *W*_6_. Each film was measured three times. The mass loss of each film was determined using the Formula (3):

(3) $$\;M(\%)=\frac{W_{5}-W_{6}}{W_{5}}\text{×}100\%\;$$

### 3.8. Materials Characterization

The morphologies of the films were characterized by a JCM-5000 SEM (JEOL, Tokyo, Japan). The particle size (diameter) of about 1000 particles were measured from the SEM images using the Nano Measurer software. Meanwhile, the energy dispersive spectra (EDS) were recorded on an Oxford INCA X-Max 250 (High Wycombe, UK). XRD was measured on a PANalytical X’Pert X-ray diffractometer (Almelo, The Netherlands). 2θ was set from 10° to 80°. The FT-IR spectra were collected on a Thermofisher Nicolet iz10 spectrometer (Framingham, MA, USA). The wavenumber was from 4000 cm^−1^ to 800 cm^−1^ and the resolution was 2 cm^−1^.

### 3.9. Inhibition Zone Assay

The assay was carried out as previously described [63]. *E. coli* and *S. aureus* were cultured at 37 °C to have an optical density (OD) at 600 nm (OD_600_) of 0.2, and were then spread on agar plates to culture with circular sterile SS/agar or AgNPs/SS/agar films (d = 0.7 cm) at 37 °C. After 12 h, the diameters of the formed inhibition zones were measured to assess the antimicrobial activity of the films.

### 3.10. Growth Curve Assay

The assay was performed as Tao’s protocol [64]. Bacteria were cultured to have an initial OD_600_ of 0.2. Then, the circular sterile SS/agar or AgNPs/SS/agar films (d = 0.7 cm) were added to culture with the bacteria at 37 °C. Bacteria (0.2 mL) were collected to measure OD_600_ at different intervals. Each film was measured in triplicate.

### 3.11. Bacterial Colony Counting Assay

The SS/agar film was soaked in AgNO_3_ solution (50 mM) at 25 °C and was then exposed to UV irradiation for 30 min to synthesize AgNPs on the composite film. The prepared film was assessed against *E. coli* for its antibacterial activity. The SS/agar film in the AgNO_3_ solution without UV irradiation was used as a control. Bacteria were cultured to have an initial OD_600_ of 0.2. Then, the circular sterile film (d = 1 cm) was added to culture with *E. coli* at 37 °C. After 6 h, 100 μL of bacterial suspension was collected and was spread on a nutrient agar plate to culture at 37 °C. After 10 h, the bacterial colonies were counted to compare the antibacterial effect of Ag (I) ions and AgNPs on *E. coli.*

### 3.12. Statistics

For all of the experiments, at least three independent tests were performed, and the data were presented as mean ± SD. The experimental data were analyzed using Student’s *t* test and a linear generalized analysis of variance (ANOVA). For the *t* test, * *p* < 0.05; ** *p* < 0.01. For the ANOVA, different lowercase letters indicate a significant difference between the groups, and the same letter means that there is no significant difference between the groups (*p* < 0.05).

## 4. Conclusions

In this work, we blended sericin with agar to prepare a SS/agar film with an enhanced mechanical performance. Then, we synthesized AgNPs in situ on the surface of the SS/agar film with the assistance of UV irradiation. The AgNPs modified composite film exhibited excellent hydrophilicity, good mechanical properties, and antimicrobial capacity toward *E. coli* and *S. aureus*. This novel sericin-based composite film with its enhanced mechanical performance and antimicrobial capability could potentially be applied in wound dressing and tissue engineering.

## Figures and Tables

**Table 1:** The diameters of the formed inhibition rings by the AgNPs/SS/agar films with different UV irradiation times.

	SS/Agar (cm)	UV 10 min (cm)	UV 30 min (cm)	UV 60 min (cm)
*E. coil*	NA	1.67 ± 0.04 ^a^	1.69 ± 0.02 ^a^	1.74 ± 0.05 ^a^
*S. aureus*	NA	1.70 ± 0.21 ^a^	1.81 ± 0.04 ^b^	2.21 ± 0.04 ^c^

NA, no inhibition ring. The superscript a, b, and c indicate that the data between the groups are statistically different. The superscript with the same letter indicates no statistical difference between the groups. *p* < 0.05.
